# The Macular Carotenoids are Associated with Cognitive Function in Preadolescent Children

**DOI:** 10.3390/nu10020193

**Published:** 2018-02-10

**Authors:** Sarah E. Saint, Lisa M. Renzi-Hammond, Naiman A. Khan, Charles H. Hillman, Janet E. Frick, Billy R. Hammond

**Affiliations:** 1 Department of Psychology, The University of Georgia, Athens, GA 30602, USA; lrenzi@uga.edu (L.M.R.-H.); jfrick@uga.edu (J.E.F.); bhammond@uga.edu (B.R.H.J.); 2 Institute of Gerontology, Department of Health Promotion and Behavior, College of Public Health, The University of Georgia, Athens, GA 30602, USA; 3 The University of Illinois at Urbana-Champaign, Department of Kinesiology and Community Health, Champaign, IL 61820, USA; nakhan2@illinois.edu; 4 Departments of Psychology and Physical Therapy, Movement & Rehabilitation Sciences, Northeastern University, Boston, MA 02115, USA; c.hillman@northeastern.edu

**Keywords:** macular pigment, lutein, cognition, children

## Abstract

The macular carotenoids lutein (L) and zeaxanthin (Z) are obtained via diet and accumulate in the central retina where they are referred to as macular pigment. The density of this biomarker (macular pigment optical density; MPOD) has been positively correlated with cognitive functioning via measures of global cognition, processing speed, and visual-spatial abilities, among others. Although improvements in cognitive function have been found in adults, much less is known about how L and Z intake may support or improve cognitive functioning during periods of rapid developmental change, such as childhood and pre-adolescence. This study examined the relationship between MPOD and cognitive functioning in 51 7–13-year-old children (51% female). MPOD was measured using heterochromatic flicker photometry (HFP) optimized for this age group. Cognitive function was assessed using the Woodcock-Johnson III (composite standard scores were obtained for Brief Intellectual Ability, Verbal Ability, Cognitive Efficiency, Processing Speed, and Executive Processes). In this sample, MPOD was significantly related to Executive Processes, *r*(47) = 0.288, *p* < 0.05, and Brief Intellectual Ability, *r*(47) = 0.268, *p* < 0.05. The relationship to Cognitive Efficiency was positive and trending but not significant, *r*(49) = 0.206, *p* = 0.074. In general, these data are consistent with those of adults showing a link between higher carotenoid status and improved cognitive functioning.

## 1. Introduction

The carotenoids lutein (L) and zeaxanthin (Z) are found in highest concentrations in dark green leafy vegetables (e.g., kale and spinach) and, when present in the diet, accumulate in the central retina where they are referred to collectively as macular pigment. In the retina, these pigments (along with their isomer, meso-zeaxanthin) serve as intraocular light filters, absorbing short-wavelength “blue” light (peak absorption at 460 nm) before it can reach the macula and damage the photoreceptors responsible for central vision. L and Z are also potent antioxidants and anti-inflammatory agents that help to protect the central nervous system from oxidative and inflammatory stress [1,2]. The brain and eye are particularly susceptible to free radical damage because they both have very high concentrations of polyunsaturated fatty acids and a high metabolic load.

Like many naturally derived compounds, the effects of L and Z on human biology are pleiotropic [3]; emerging research, for instance, has demonstrated a relationship between the macular carotenoids and cognitive performance in adults (see [4] for a review). There is reason to believe that these molecules may also be important for cognitive development in early life, but the relationship between L and Z status, measured directly in the central nervous system, and cognitive performance has only recently been examined in children [5,6,7].

An effect of L and Z on the developing retina/brain is biologically feasible [8]. L is the predominant carotenoid in the developing fetal and infant brain, despite relatively low dietary intake, and makes up 59% of the carotenoids in the infant brain [9] compared to 34% in geriatric adults [10]. It has been suggested that such high concentrations of L in the developing brain are an indication that it may be necessary during periods of rapid neural development [8,11,12]. Development is a time characterized by increased vulnerability to oxidative and inflammatory stress [13] and children tend to have significantly lower intake of L and Z compared to adults [14]. When studied in model cell cultures, carotenoids have been shown to promote the formation of gap junctions between cells [15]. Promoting gap junction communication would allow neurons to communicate laterally via direct ion exchange. This may improve cell-to-cell communication and could lead to faster and more efficient processing within the visual system, as well as throughout the central nervous system (i.e., the neural efficiency hypothesis [16,17]). Evidence showing that L and Z supplementation increases visual processing speed [18,19] is consistent with this possibility.

A number of studies have shown that higher L and Z predict better cognitive outcomes [5,7,20,21,22,23]. For example, low macular pigment optical density (MPOD) in a sample of older adults was associated with significantly lower performance on global measures of cognitive functioning (mini-mental status exam (MMSE) and Montreal Cognitive Assessment (MoCA)), as well as prospective memory and processing speed tasks [21]. MPOD has also been associated with attention and cognitive flexibility (as assessed by task switching), as well as visual memory and learning (paired associate learning task) in both healthy adults and those with retinal disease [24]. Older adults with higher MPOD exhibited less brain activation to complete a verbal learning task in a recent fMRI study [25].

Intervention trials with L and Z have yielded similar findings. For example, healthy older women who were supplemented with L, docosahexaeonic acid (DHA, an omega-3 fatty acid), or a combination of L and DHA showed improved verbal fluency in all three groups, as well as improvements in performance on several delayed recall memory tests in the L + DHA group [20]. A recent placebo-controlled trial involving older adults found that supplementation with L and Z improved performance on measures of complex attention and executive functions (i.e., cognitive flexibility) [23].

Similar observations have been made even in the very young. For example, infant recognition memory, tested using an event-related potential (ERP) oddball paradigm, has been positively associated with the amount of L and choline in mother’s breastmilk [26]. MPOD, measured in preadolescent children, has been shown to associate positively with educational achievement (math and written comprehension) [5], aspects of relational memory on a spatial reconstruction task [7], as well as cognitive control performance and ERP outcomes on an attentional inhibition task [6].

These past studies have shown that MPOD can predict academic performance (math and written comprehension) and lab-based cognitive outcomes in preadolescent children. Whether MPOD can also predict outcomes on standardized tests of cognition, as it has been shown to do in adults [27], has yet to be determined. The present study tests the hypothesis that MPOD will positively correlate with performance on standardized cognitive assessments of global intelligence, verbal ability, cognitive efficiency, processing speed, and executive processes using the standardized Woodcock Johnson III Tests of Cognitive Abilities [28].

## 2. Materials and Methods

### 2.1. Participants

Fifty-four children (45.5% female) were recruited from the Athens, Georgia community. Data from three of these children were excluded from all analyses for the following reasons: (a) *n* = 2 participants had diagnoses that made testing (cognitive and/or MPOD) challenging (i.e., sensory processing disorder, ADHD), and (b) *n* = 1 participant was an outlier on the cognitive outcomes and complained of excessive fatigue (not characteristic of other subjects) during testing. The final sample consisted of 51 children (49% female). Children ranged in age from 7 to 13 and were largely white (non-Hispanic; 76.5%) and from well-educated families (90.2% of children had at least one parent with some level of post-secondary education). See Table 1 for complete demographic information. All participants had normal (or corrected-to-normal) vision while completing the tasks. Informed consent was obtained prior to participation from each participant’s accompanying parent/guardian, in addition to the participant’s assent, which was given verbally and/or in writing after an age-appropriate discussion of the study and what is meant by “voluntary participation”. All study activities were carried out in accordance with the Declaration of Helsinki, and the study protocol was approved by the Ethics Committee of the University of Georgia (Study ID: 2013100730).

### 2.2. Measures

#### 2.2.1. Macular Pigment Optical Density (MPOD)

MPOD was measured using customized heterochromatic flicker photometry (HFP) via a Macular Densitometer™ (Macular Metrics Corporation, Rehoboth, MA, USA) as described in [29]. Measurement of MPOD in children using HFP has been demonstrated to be possible with a moderate degree of reliability (Cronbach’s α = 0.72) [30]. The standard one-degree and 460 nm test stimulus was used and the procedure largely followed that described in [30]. One difference from [30] in the present study is that testing was confined to only two experimenters who had extensive experience working with children, and the method of adjustment was used.

#### 2.2.2. Cognitive Testing

Selected tests from the Woodcock-Johnson III (WJ-III) Tests of Cognitive Abilities [28] were used to assess children’s cognitive functioning. The WJ-III is a norm-referenced set of tests designed to measure intellectual abilities in 2- to 90+-year-olds. The WJ-III was designed using the Cattell-Horn-Carroll Theory of Cognitive Abilities and has been standardized on over 8000 individuals who are representative of the demographics and communities of the general United States population [31]. All subtests of interest for this study have median reliability scores of 0.8 or higher, with the exception of the Planning subtest (median reliability = 0.75) [31]. All Cognitive Performance Composite scores of interest have median reliability scores of 0.9 or higher [31].

Participants completed the following WJ-III standard battery subtests: Verbal Comprehension, Concept Formation, Visual Matching 2, Numbers Reversed, Decision Speed, Planning, and Pair Cancellation. These subtests were chosen so that the following cognitive performance cluster scores could be calculated: Brief Intellectual Ability (BIA), Verbal Ability, Cognitive Efficiency, Processing Speed, and Executive Processes. Time constraints prevented the administration of the entire WJ-III standard battery, therefore these cluster scores were selected as being most likely to be related to levels of L and Z in the CNS based on previous work with adults, children, and infants [20,21,22,23,26].

One trained experimenter was responsible for testing all of the participants to reduce potential inter-rater reliability confounds. Participants were allowed to take breaks between subtests, and the order in which subtests were completed was altered as needed to maintain attention. For example, the Concept Formation subtest is particularly challenging, and participants frequently feel cognitively fatigued by the time that test is administered. On occasion, that subtest was moved to a later point in the testing session to allow participants to recover their attention during the more hands-on tasks (e.g., Visual Matching 2, Decision Speed, Planning, and Pair Cancellation) before attempting the more challenging Concept Formation task. Completion of these WJ-III subtests took 90 min to two hours. The decision to alter the order of subtest administration was made during the testing session based on experimenter judgment of the participant’s level of fatigue.

### 2.3. Statistical Analyses

Statistical analyses were performed using SPSS version 24 (IBM, Armonk, NY, USA) and α = 0.05 was used as the cutoff value for statistical significance. Bivariate correlations were calculated between MPOD and all cognitive variables, and one-tailed test values are reported (unless otherwise specified) given the directional nature of all a priori hypotheses (i.e., higher MPOD is associated with higher cognitive functioning). Partial correlations were calculated when necessary to control for sex or age differences. The distributional shapes of the MPOD and cognitive composite score variables were examined and found to meet the assumption of normality (*S*-*W* ≥ 0.967, *df* = 49, *p* ≥ 0.181 for all variables).

## 3. Results

The standard scores for the WJ-III Brief Intellectual Ability, Processing Speed, Cognitive Efficiency, and Executive Processes composite measures were used to assess specific components of cognitive functioning to control for age differences among participants. Four of the children tested were born prematurely (<37 weeks gestation). Given that prematurity has been linked to deficits in processing speed and academic achievement into adolescence [32,33], independent-samples *t*-tests were conducted to determine whether any differences in cognitive or visual performance existed based on prematurity. No significant differences were found for either measure (visual or cognitive), therefore these children were kept in the data set. Sex differences in WJ-III performance were detected for the Processing Speed composite score and Visual-Auditory Learning subtest; female participants demonstrated higher performance on both measures, *t*(49) = −2.795, *p* = 0.007 and *t*(49) = −2.119, *p* = 0.039 (two-tailed), respectively. Sex differences were not detected for MPOD or any other cognitive variables.

Two participants (7- and 10-years-old) were unwilling to complete the Concept Formation subtest of the WJ-III, which is required to calculate the BIA and Executive Processes composite scores, resulting in missing data for those variables. Additionally, one 7-year-old was unwilling to complete the Spatial Relations subtest. Final sample size and descriptive statistics for all measures can be found in Table 2.

**Figure 1:**
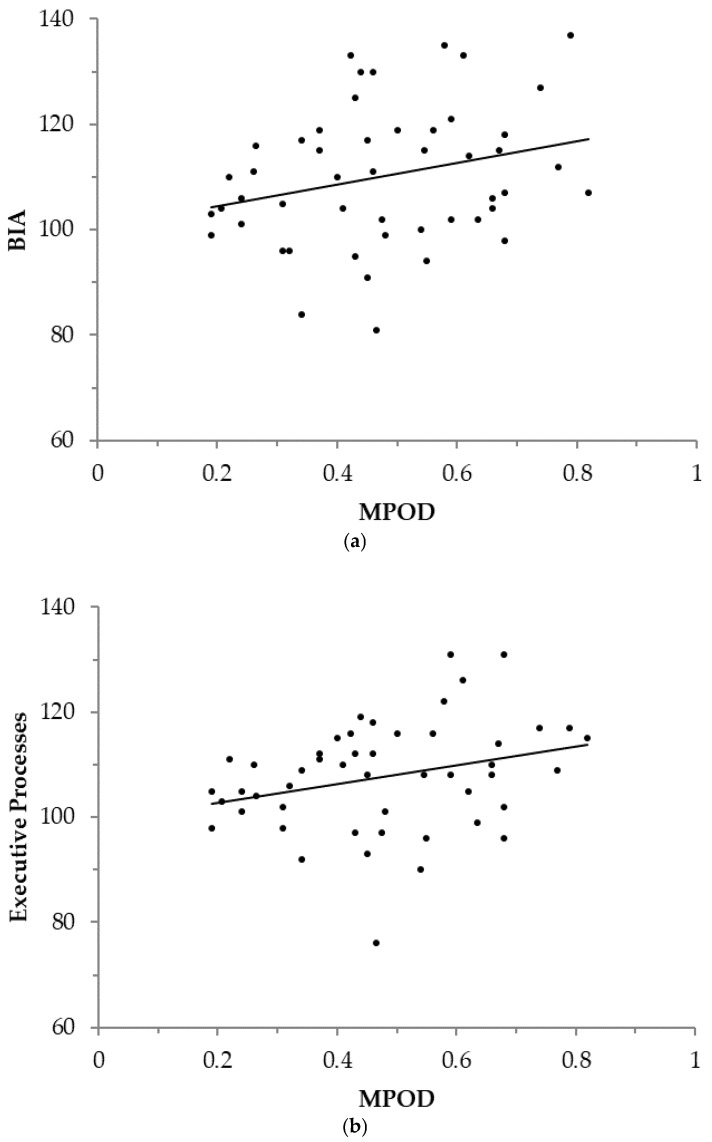
The relationship between macular pigment optical density (MPOD) and the following WJ-III Cognitive Composite scores: (**a**) Brief Intellectual Ability (BIA), regression line constant = 100.295, β = 20.523, *R*^2^(47) = 0.072, and (**b**) Executive Processes, regression line constant = 99.234, β = 17.705, *R*^2^(47) = 0.083. Standard scores were used for all cognitive variables to control for age. Note: Regression equations were calculated with MPOD predicting cognitive performance, though it should be noted that the correlational design of the present study does not allow for causal interpretations.

MPOD was significantly related to global intelligence (Brief Intellectual Ability; BIA) and Executive Processes composite scores (age-normed; see Table 3 and Figure 1). In addition, the relationship between MPOD and Cognitive Efficiency approached significance, *r*(49) = 0.206, *p* = 0.074 (one-tailed). The Verbal Learning and Processing Speed measures were not significantly related to MPOD (see Table 3; both *p*s > 0.10, one-tailed).

**Figure 2:**
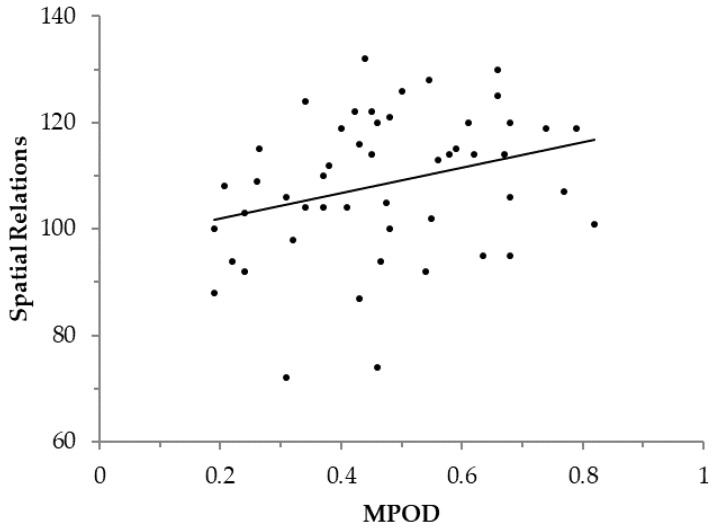
The relationship between macular pigment optical density (MPOD) and the WJ-III Spatial Relations subtest, regression line constant = 97.073, β = 24.081, *R*^2^(48) = 0.090. Standard scores were used for Spatial Relations variable to control for age. Note: Regression equation was calculated with MPOD predicting cognitive performance, though it should be noted that the correlational design of the present study does not allow for causal interpretations.

Exploratory analyses were performed using MPOD and two WJ-III subtests that were not included in the calculation of the cluster scores reported above. Performance on the Spatial Relations subtest (a measure of visual-spatial thinking abilities) was positively related to MPOD, *r*(48) = 0.299, *p* = 0.035 (two-tailed; see Figure 2), while the partial correlation controlling for sex between MPOD and performance on the Visual-Auditory Learning subtest (a measure of paired-associate learning) did not reach significance, *r*(48) = 0.236, *p* = 0.099 (two-tailed; see Table 4).

## 4. Discussion

The present study tested whether MPOD, a marker of macular pigments L and Z concentration in the retina, related to standardized measures of cognitive functioning in pre-adolescent children. MPOD correlates highly with levels of lutein in the brain [34] and is therefore considered a reliable biomarker of overall central nervous system carotenoid status. Our findings further demonstrate that the ability to measure these carotenoids directly within neural tissue is a unique and powerful approach to assess the role these phytochemicals play in that very tissue: MPOD was positively related to global intelligence (BIA), executive functioning, and visuo-spatial thinking abilities in this sample of preadolescent children. In addition, the relationship between MPOD and cognitive efficiency approached significance.

These cognitive processes are founded in networks involving the frontal and parietal cortices, with input from the occipital cortex (as well as other regions), and the frontal and occipital cortices are brain regions that contain particularly high concentrations of L and Z, when they are present in diet [34]. Our results are in general agreement with data collected from adult and aging populations, as well as recent studies of other samples of preadolescent children [5,6,7,20,21,22,23]. Higher MPOD has been associated with higher cognitive performance in a number of areas. For example, MPOD relates to measures of global cognitive functioning and executive functioning in older adults [21,23]. These types of cognitive effects have also been manifest when using neuroimaging tools. For example, studies using fMRI have shown that higher MPOD is related to greater efficiency in the form of reduced brain activation during task execution in adults [25], and EEG work has demonstrated better cognitive control in both young children and adults [6,22].

Children’s scores on the WJ-III Processing Speed composite measure were not related to MPOD in the present study. The tests that make up the WJ-III Processing Speed Composite score require children to circle items on a page (given various rules, such as “circle the two items in each row that are most alike” or “circle the two numbers in each row that are the same”) as fast as they can until they reach the end or three minutes has passed. Despite the “speed” designation, this test is unlike other measures of processing speed, such as critical flicker fusion frequency (CFF), that are significantly related to MPOD in young adults [16,17,18]. Tasks such as CFF are atomistic in nature. They are limited by the transmission speed of neurons within the visual cortex and do not reflect the additional processing necessary for decision-making and understanding relationships between items in categories, which are largely frontal lobe phenomena [35].

These results highlight the importance of diet in supporting cognitive health in preadolescent children. It is likely that the participants tested in our study (similar to those tested in [30]) may reflect less deficiency than is often seen in the average American diet given the sample was collected in a large public university-based community (our average MPOD was relatively high). The latest National Health and Nutrition Examination Survey (NHANES) fruit and vegetable intake report reveals that dark green vegetables were consumed by only 10.7% of US children between the ages of 6–11 on a given day in 2009–2010 [36]. This dietary behavior is reflected in relatively low average intake of L and Z (about three times lower than adults) [14] and suggest that this dietary behavior may be worsening over time. Children under the age of 12 decreased their yearly vegetable intake between 2009 and 2015 by 12 servings per capita [37]. The present study was not designed to establish a causal relationship between MPOD (or L and Z intake) and cognition, but given the cross-sectional relationships between L and Z with cognitive outcomes in children, future studies should attempt to do so. This could be done by increasing L and Z intake in children via supplementation or dietary interventions to determine whether children also exhibit the same positive benefits that adult supplementation studies have demonstrated [18,20,23].

## Figures and Tables

**Table 1:** Descriptive statistics for all participants included in analyses.

	*N* (%)
**Age (years)**	
7	16 (31.4)
8	5 (9.8)
9	9 (17.6)
10	6 (11.8)
11	8 (15.7)
12	6 (11.8)
13	1 (2.0)
**Sex**	
Male	26 (51.0)
Female	25 (49.0)
Race	
White (Non-Hispanic)	39 (76.5)
Hispanic	1 (2.0)
>1 Race Listed	11 (21.6)
**Parent Highest Education**	
High School or less	3 (5.9)
College Degree (AS, BS, BA)	18 (35.3)
Graduate Degree	28 (54.9)

**Table 2:** Descriptive statistics for all measures.

	Mean	SD	Range	*N*
**MPOD**	**0.476**	**0.167**	**0.190–0.820**	**51**
**Cognitive Measures (All Standard Scores)**				
*WJ-III Composite Scores*				
Brief Intellectual Ability (BIA)	110.10	13.012	81–137	49
Verbal Ability	112.41	12.420	89–144	51
Cognitive Efficiency	104.02	15.909	65–132	51
Processing Speed	100.10	17.258	75–151	51
Executive Processes	107.69	10.453	76–131	49
*Select WJ-III Subtests*				
Visual-Auditory Learning	100.84	13.249	75–132	51
Spatial Relations	108.48	13.526	72–132	50

Note: MPOD = Macular Pigment Optical Density. SD: standard deviation.

**Table 3:** Correlations among MPOD and WJ-III cognitive cluster scores. All correlations are bivariate with the exception of Processing Speed, which was calculated as a partial correlation controlling for sex, given that sex differences were evident in the Processing Speed variable.

	BIA	Verbal Ability	Cognitive Efficiency	Processing Speed	Executive Processes
MPOD	0.268 * (*N* = 49)	0.159 (*N* = 51)	0.206 ^†^ (*N* = 51)	0.099 (*N* = 51)	0.288 * (*N* = 49)

* *p* < 0.05, ^†^ *p* ≤ 0.10 (one-tailed). Note: MPOD = Macular Pigment Optical Density. BIA = Brief Intellectual Ability. The number of subjects who completed each set of measures is reported in parentheses below each correlation.

**Table 4:** Correlations among MPOD and two WJ-III cognitive subtests of interest. The correlation between MPOD and Visual-Auditory Learning was calculated as a partial correlation controlling for sex, given that sex differences were evident in that cognitive subtest.

	Spatial Relations	Visual-Auditory Learning
MPOD	0.299 * (*N* = 50)	0.236 ^†^ (*N* = 51)

* *p* < 0.05, ^†^ *p* ≤ 0.10 (two-tailed). Note: MPOD = Macular Pigment Optical Density. The number of subjects who completed each set of measures is reported in parentheses below each correlation.

## References

[1] Stahl W., Sies H. (2003). Antioxidant activity of carotenoids. Mol. Asp. Med..

[2] Ozawa Y., Sasaki M., Takahashi N., Kamoshita M., Miyake S., Tsubota K. (2012). Neuroprotective Effects of Lutein in the Retina. Curr. Pharm. Des..

[3] Hammond B. Lutein’s Influence on Neural Processing Speed. Proceedings of the 114th Abbott Nutrition Research Conference, Cognition and Nutrition.

[4] Jia Y.P., Sun L., Yu H.S., Liang L.P., Li W., Ding H., Song X.B., Zhang L.J. (2017). The pharmacological effects of lutein and zeaxanthin on visual disorders and cognition diseases. Molecules.

[5] Barnett S.M., Khan N.A., Walk A.M., Raine L.B., Moulton C., Cohen N.J., Kramer A.F., Hammond B.R., Renzi-Hammond L., Hillman C.H. (2017). Macular pigment optical density is positively associated with academic performance among preadolescent children. Nutr. Neurosci..

[6] Walk A.M., Khan N.A., Barnett S.M., Raine L.B., Kramer A.F., Cohen N.J., Moulton C.J., Renzi-Hammond L.M., Hammond B.R., Hillman C.H. (2017). From neuro-pigments to neural efficiency: The relationship between retinal carotenoids and behavioral and neuroelectric indices of cognitive control in childhood. Int. J. Psychophysiol..

[7] Hassevoort K.M., Khazoum S.E., Walker J.A., Barnett S.M., Raine L.B., Hammond B.R., Renzi-Hammond L.M., Kramer A.F., Khan N.A., Hillman C.H. (2017). Macular Carotenoids, Aerobic Fitness, and Central Adiposity Are Associated Differentially with Hippocampal-Dependent Relational Memory in Preadolescent Children. J. Pediatr..

[8] Hammond B.R. (2012). The Dietary Carotenoids Lutein and Zeaxanthin in Pre-and-Postnatal Development. Funct. Food Rev..

[9] Vishwanathan R., Kuchan M.J., Sen S., Johnson E.J. (2014). Lutein and preterm infants with decreased concentrations of brain carotenoids. J. Pediatr. Gastroenterol. Nutr..

[10] Johnson E.J., Vishwanathan R., Johnson M.A., Hausman D.B., Davey A., Scott T.M., Green R.C., Miller L.S., Gearing M., Woodard J. (2013). Relationship between serum and brain carotenoids, α-tocopherol, and retinol concentrations and cognitive performance in the oldest old from the Georgia centenarian study. J. Aging Res..

[11] Hammond B.R. (2008). Possible role for dietary lutein and zeaxanthin in visual development. Nutr. Rev..

[12] Johnson E.J. (2014). Role of lutein and zeaxanthin in visual and cognitive function throughout the lifespan. Nutr. Rev..

[13] Holt E.M., Steffen L.M., Moran A., Basu S., Steinberger J., Ross J.A., Hong C.P., Sinaiko A.R. (2009). Fruit and Vegetable Consumption and Its Relation to Markers of Inflammation and Oxidative Stress in Adolescents. J. Am. Diet. Assoc..

[14] Johnson E.J., Maras J.E., Rasmussen H.M., Tucker K.L. (2010). Intake of Lutein and Zeaxanthin Differ with Age, Sex, and Ethnicity. J. Am. Diet. Assoc..

[15] Stahl W., Sies H. (2001). Effects of carotenoids and retinoids on gap junctional communication. BioFactors.

[16] Hammond B.R., Wooten B.R. (2005). CFF thresholds: Relation to macular pigment optical density. Ophthalmic Physiol. Opt..

[17] Renzi L.M., Hammond B.R. (2010). The relation between the macular carotenoids, lutein and zeaxanthin, and temporal vision. Ophthalmic Physiol. Opt..

[18] Bovier E.R., Renzi L.M., Hammond B.R. (2014). A double-blind, placebo-controlled study on the effects of lutein and zeaxanthin on neural processing speed and efficiency. PLoS ONE.

[19] Bovier E.R., Hammond B.R. (2015). A randomized placebo-controlled study on the effects of lutein and zeaxanthin on visual processing speed in young healthy subjects. Arch. Biochem. Biophys..

[20] Johnson E., McDonald K., Caldarella S., Chung H.-Y., Troen A., Snodderly D. (2008). Cognitive findings of an exploratory trial of docosahexaenoic acid and lutein supplementation in older women. Nutr. Neurosci..

[21] Feeney J., Finucane C., Savva G.M., Cronin H., Beatty S., Nolan J.M., Kenny R.A. (2013). Low macular pigment optical density is associated with lower cognitive performance in a large, population-based sample of older adults. Neurobiol. Aging.

[22] Walk A.M., Edwards C.G., Baumgartner N.W., Curran M.R., Covello A.R., Reeser G.E., Hammond B.R., Renzi L.M., Khan N.A. (2017). The Role of Retinal Carotenoids and Age on Neuroelectric Indices of Attentional Control among early to middle-aged adults. Front. Aging Neurosci..

[23] Hammond B.R., Stephen Miller L., Bello M.O., Lindbergh C.A., Mewborn C., Renzi-Hammond L.M. (2017). Effects of lutein/zeaxanthin supplementation on the cognitive function of community dwelling older adults: A randomized, double-masked, placebo-controlled trial. Front. Aging Neurosci..

[24] Kelly D., Coen R.F., Akuffo K.O., Beatty S., Dennison J., Moran R., Stack J., Howard A.N., Mulcahy R., Nolan J.M. (2015). Cognitive function and its relationship with macular pigment optical density and serum concentrations of its constituent carotenoids. J. Alzheimer’s Dis..

[25] Lindbergh C.A., Mewborn C.M., Hammond B.R., Renzi-Hammond L.M., Curran-Celentano J.M., Miller L.S. (2016). Relationship of lutein and zeaxanthin levels to neurocognitive functioning: An fMRI study of older adults. J. Int. Neuropsychol. Soc..

[26] Cheatham C.L., Sheppard K.W. (2015). Synergistic effects of human milk nutrients in the support of infant recognition memory: An observational study. Nutrients.

[27] Renzi L.M., Dengler M.J., Puente A., Miller L.S., Hammond B.R. (2014). Relationships between macular pigment optical density and cognitive function in unimpaired and mildly cognitively impaired older adults. Neurobiol. Aging.

[28] Woodcock R.W., McGrew K.S., Mather N. (2001). Woodcock-Johnson III Tests of Cognitive Abilities.

[29] Wooten B.R., Hammond B.R., Land R.I., Snodderly D.M. (1999). A practical method for measuring macular pigment optical density. Investig. Ophthalmol. Vis. Sci..

[30] McCorkle S., Raine L., Hammond B., Renzi-Hammond L., Hillman C., Khan N. (2015). Reliability of heterochromatic flicker photometry in measuring macular pigment optical density among preadolescent children. Foods.

[31] Mather N., Woodcock R.W. (2001). Examiner’s Manual: Woodcock-Johnson III Tests of Cognitive Abilities.

[32] Rose S., Feldman J., Jankowski J., Van Rossem R. (2011). Basic information processing abilities at 11years account for deficits in IQ associated with preterm birth. Intelligence.

[33] Rose S., Feldman J., Jankowski J. (2012). Implications of infant cognition for executive functions at age 11. Psychol. Sci..

[34] Vishwanathan R., Neuringer M., Snodderly D.M., Schalch W., Johnson E.J. (2013). Macular lutein and zeaxanthin are related to brain lutein and zeaxanthin in primates. Nutr. Neurosci..

[35] Mishkin M., Weiskrantz L. (1959). Effects of cortical lesions in monkeys on critical flicker frequency. J. Comp. Physiol. Psychol..

[36] Nielsen S.J., Rossen L.M., Harris D.M., Ogden C.L. (2014). Fruit and Vegetable Consumption of U.S. Youth, 2009–2010.

[37] Produce for Better Health Foundation (2015). State of the Plate, 2015 Study on America’s Consumption of Fruit and Vegetables. http://www.pbhfoundation.org.

